# Determinants of quality of life in adults with type 1 and type 2 diabetes

**DOI:** 10.1186/1477-7525-9-115

**Published:** 2011-12-19

**Authors:** Ikuyo Imayama, Ronald C Plotnikoff, Kerry S Courneya, Jeffrey A Johnson

**Affiliations:** 1 Centre for Health Promotion Studies, School of Public Health, University of Alberta, (116 Street and 85 Avenue), Edmonton, (T6G 2B3), Canada; 2 School of Education, University of Newcastle, Callaghan, (2308), Australia; 3 Faculty of Physical Education, University of Alberta, (116 Street and 85 Avenue), Edmonton, (T6G 2B3), Canada; 4 School of Public Health, University of Alberta, (116 Street and 85 Avenue), Edmonton, (T6G 2B3), Canada

**Keywords:** quality of life, health-related quality of life, life satisfaction, type 1 diabetes, type 2 diabetes, adults with diabetes

## Abstract

**Background:**

Limited evidence exists on the determinants of quality of life (QoL) specific to adults with type 1 diabetes (T1D). Further, it appears no study has compared the determinants of QoL between T1D and type 2 diabetes (T2D) groups. The objectives of this study were to examine: (1) determinants of QoL in adults with T1D; and, (2) differences in QoL determinants between T1D and T2D groups.

**Methods:**

The Alberta Longitudinal Exercise and Diabetes Research Advancement (ALEXANDRA) study, a longitudinal study of adults with diabetes in Alberta, Canada. Adults (18 years and older) with T1D (N = 490) and T2D (N = 1,147) provided information on demographics (gender, marital status, education, and annual income), personality (activity trait), medical factors (diabetes duration, insulin use, number of comorbidities, and body mass index), lifestyle behaviors (smoking habits, physical activity, and diet), health-related quality of life (HRQL) and life satisfaction. Multiple regression models identified determinants of HRQL and life satisfaction in adults with T1D. These determinants were compared with determinants for T2D adults reported in a previous study from this population data set. Factors significantly associated with HRQL and life satisfaction in either T1D or T2D groups were further tested for interaction with diabetes type.

**Results:**

In adults with T1D, higher activity trait (personality) score (β = 0.28, p < 0.01), fewer comorbidities (β = **-**0.27, p < 0.01), lower body mass index (BMI)(β = **-**0.12, p < 0.01), being a non-smoker (β = **-**0.14, p < 0.01), and higher physical activity levels (β = 0.16, p < 0.01) were associated with higher HRQL. Having a partner (β = 0.11, p < 0.05), high annual income (β = 0.16, p < 0.01), and high activity trait (personality) score (β = 0.27, p < 0.01) were significantly associated with higher life satisfaction. There was a significant age × diabetes type interaction for HRQL. The T2D group had a stronger positive relationship between advancing age and HRQL compared to the T1D group. No interaction was significant for life satisfaction.

**Conclusions:**

Health services should target medical and lifestyle factors and provide support for T1D adults to increase their QoL. Additional social support for socioeconomically disadvantaged individuals living with this disease may be warranted. Health practitioners should also be aware that age has different effects on QoL between T1D and T2D adults.

## Background

More than 180 million people worldwide have diabetes mellitus, and the number of diabetes patients is estimated to double by 2030 [1]. The increasing trend of diabetes has been reported for both type 1 diabetes (T1D) [2-4] and type 2 diabetes (T2D) populations [5,6].

Diabetes has detrimental effects on health outcomes including quality of life (QoL) outcomes [7] and studies have shown significant negative associations for health-related quality of life (HRQL), one specific aspect of QoL, with its prognosis [8-10]. Thus, further understanding the determinants of HRQL and QoL among individuals with diabetes could guide tailored and targeted intervention strategies to improve these outcomes for this population group.

We examined personal, medical and lifestyle determinants of HRQL and life satisfaction in adults with type 2 diabetes in a previous study [11] and found older age, higher income, higher score on activity (personality) trait, not using insulin, having fewer comorbidities, lower BMI, being a non-smoker, and a higher physical activity level were significantly associated with better HRQL in adults with T2D. Age, gender, marital status, income, activity trait, insulin, comorbidities, higher BMI, smoking, and higher general diet score were significantly associated with life satisfaction.

As for T1D, although several studies have examined determinants of HRQL in adolescents and young adults with T1D [12-17], only a few studies have examined the determinants of HRQL and QoL in adults with T1D. One study that examined 397 adults with T1D, reported that female gender, lower income, longer diabetes duration, diabetes complications, experiencing more than one episode of hypoglycemia per month, and low physical activity levels were associated with poor HRQL [18]. Another study found female gender, obesity, diabetes complication and comorbidities were associated with lower HRQL, among 784 T1D adults [19]. Further Parkerson and colleagues [20] found that marital status, social relationships, and comorbidities were associated with HRQL among 170 T1D adults [20].

Despite aetiological differences between T1D and T2D [21-23], differences in levels of HRQL and QoL as well as their determinants between the two diabetes types have not been thoroughly investigated in adults with diabetes. Jacobson and colleagues [24] compared HRQL scores between 240 adults with T1D or T2D, and identified higher HRQL in T2D after adjusting for demographic factors (i.e., age, marital status and education), diabetes complications, and diabetes duration. Another study compared levels of three HRQL measures in adults (T1D, N = 236; T2D, N = 889) and found no differences in EQ-5D and QoL-DN scores between the two samples, but a higher global health profile (SF-36) score in the T2D group was reported [25]. Finally, in two studies on youth with diabetes, HRQL was lower among T2D individuals compared to those with T1D [26,27].

From the above, it is apparent that a limited number of studies have investigated the determinants of HRQL in adults with T1D. Further, despite the aetiological and HRQL differences between the two diabetes types, it appears limited research has specifically examined the differences in determinants of HRQL and QoL between T1D and T2D adults. The previous literature on diabetes populations has focused primarily on HRQL, while evidence on QoL (a broader concept which includes general well-being and life satisfaction dimensions) is sparse. Moreover, while the above studies have examined the differences in the relationships of demographic factors, [24,27] medical factors (e.g., diabetes duration, complications) [24,25,27,28] with HRQL between the two diabetes groups, to our knowledge, no study has tested models consisting of personality and lifestyle factors to understand the differences in the determinants of HRQL and QoL between these two diabetes groups. In particular, due to the important role that lifestyle behaviors play on the etiology of diabetes management [23] and on improved HRQL [29], it is therefore important to include lifestyle behaviors in multivariate models to examine the determinants of HRQL and QoL between the two diabetes groups.

Therefore, the objectives of this study were to use a comprehensive model [11] to examine: (1) the determinants of HRQL and QoL (life satisfaction) in adults with T1D; and, (2) the interaction effects of diabetes type (i.e., T1D/T2D) on significant determinants of HRQL and QoL in the combined T1D and T2D group. In regards to the first study objective, we hypothesized that personal factors (age, gender, personality), medical factors (duration of diabetes, number of comorbidities, BMI) and lifestyle factors (physical activity) are associated with HRQL and life satisfaction in adults with type 1 diabetes. Due to the exploratory nature of second study objective, no specific *a priori *hypotheses were made for the variables (personal, medical, lifestyle and the interaction effects of diabetes type) examined in the multivariate models.

## Methods

The Alberta Longitudinal Exercise and Diabetes Research Advancement (ALEXANDRA) study was a population-based, longitudinal study of physical activity determinants in adults with diabetes in Alberta, Canada. The baseline data collection commenced in May 2002. The study procedures, response rates, and measures are explained elsewhere [30]. In brief, the ALEXANDRA study assessed factors related to physical activity in adults (18 years and older) with diabetes. Baseline assessments were completed by 2,319 individuals with diabetes and 1,662 (510 with T1D and 1,152 with T2D) completed the 6-month assessment. The data from the 6-month assessment were used for this study. The study protocol was reviewed by the University of Alberta Health Research Ethics Board. All participants completed written informed consent.

The determinants of HRQL and life satisfaction in the T2D group from the ALEXANDRA Study have been reported elsewhere [11]. This paper reports the determinants of HRQL and life satisfaction in the ALEXANDRA study T1D group, and compares the determinants of these outcomes between the T1D and T2D groups.

### Measures

Self-report questionnaires were used to collect data on all study variables. Demographic factors (i.e., age, gender, marital status, education, and income) were assessed using identical measures from the Statistics Canada 2001 census [31]. Personality (i.e., activity trait) was measured by Saucier and Ostendorf's [32] 5-item unipolar activity trait markers (i.e., unadventurous, rambunctious, competitive, unenergetic and active), and the mean scores of the five items were used.

#### Medical factors

Diabetes type, duration of diabetes, insulin use, presence of comorbidities (angina, heart attack, stroke, high blood cholesterol, and high blood pressure), and BMI (kg/m^2^) were assessed, and the total number of comorbidities for each individual was calculated (score range from 0 to 5).

#### Lifestyle factors

Smoking habits were assessed by asking current smoking behavior [33]. Physical activity was measured by a modified version of the Godin Leisure-Time Exercise Questionnaire (GLTEQ) [34-36]. Total weekly minutes of moderate and vigorous physical activity were used [37]. Three diet behaviors (i.e., general and specific diet, and carbohydrate spacing) were assessed by the revised version of Diabetes Self-Care Activities measure [38].

#### Quality of life variables

HRQL was assessed by a single-item question: "In general, compared to other persons your age, would you say your health is poor/fair/good/very good/excellent." The response score of 1 (poor) to 5 (excellent) was calibrated into value of 0 (poor) to 100 (excellent) [39]. The use of a single item question to assess HRQL has been recommended in large population surveys [40,41]. The 5-item Satisfaction with Life Scale was used to measure life satisfaction [42].

### Data analysis

The characteristics between T1D and T2D groups were compared using t-tests and Chi-square analyses. For the T1D sample, we tested four models consisting of personal (Model 1), medical (Model 2), lifestyle factors (Model 3), and all variables (Model 4) to explain HRQL and life satisfaction [11]. Model 1 included demographics and personality. Model 2 included duration of diabetes, a number of comorbid conditions and BMI. Model 3 consisted of smoking habits, physical activity and three dietary behaviors. Model 4 included all variables of Model 1, 2 and 3. A multiple regression analysis was used to identify variables significantly associated with HRQL and life satisfaction in the T1D group and variances explained by the models.

Variables significantly associated with HRQL and life satisfaction in either the T1D or T2D groups were included and further tested for interaction effects between the two diabetes type groups. Interaction variables were created by multiplying independent variables with diabetes type. To avoid collinearity among variables, residuals of the interaction variables were used for the analysis [43]. All analyses were performed by SPSS for Windows 15.0.

## Results

### Sample characteristics of adults with type 1 diabetes

Table 1 displays the characteristics of study sample by diabetes type. The T1D group (51.5 ± 16.4 years) were younger compared with T2D group (63.7 ± 11.4 years, p < 0.0001). The percent of female was higher among T1D group (53.1%) compared to T2D group (47.3%, p = 0.03). More participants in the T2D group had a college degree and higher (43.7%) compared to T1D group (34.9%, p = 0.001). There were no differences in marital status and personality scores (activity trait) between the two diabetes groups. The mean diabetes duration was longer in T1D group (21.6 ± 12.8 years) than in T2D group (11.2 ± 12.8 years). Individuals with T2D had more comorbidities and higher BMI compared to those with T1D (p < 0.0001). There were no differences in smoking habits and physical activity levels between the two groups. T1D group had higher general diet and spacing carbohydrates scores than T2D group (p ≤ 0.01), while the specific diet scores were higher among T2D group (vs. T1D group, p = 0.05). The mean (SD) of HRQL scores were 54.8 ± 26.9 in T1D group and 54.7 ± 25.7 in T2D group. The life satisfaction scores for T1D and T2D groups were 16.2 ± 4.3 and 16.6 ± 4.3, respectively. There were no differences in HRQL and life satisfaction scores between the two diabetes groups.

**Table 1:** Characteristics of type 1 and type 2 diabetes samples

	Type 1 diabetes (N = 490)		Type 2 diabetes (N = 1147)		
	Mean (SD)	N (%)	Mean (SD)	N (%)	P value*
**Demographic factors**					
Age (years)	51.5 (16.4)		63.7 (11.4)		< 0.0001
Gender					
Male		230 (46.9)		605 (52.7)	0.03
Female		260 (53.1)		542 (47.3)	
Marital status					0.11
No partner		125 (25.5)		251 (21.9)	
Have partner		365 (74.5)		896 (78.1)	
Education					0.001
No college degree		276 (56.3)		747 (65.1)	
College degree and higher		214 (43.7)		400 (34.9)	
Annual income					< 0.0001
< $20,000		50 (10.2)		157 (13.7)	
$20,000-39,999		112 (22.9)		330 (28.8)	
$40,000-59,999		105 (21.4)		293 (25.5)	
$60,000-79,999		109 (22.2)		172 (15.0)	
$80,000-99,999		43 (8.8)		89 (7.8)	
$100,000 <		71 (14.5)		106 (9.2)	
Activity (personality) trait (score 1-5)	3.3 (0.6)		3.2 (0.6)		0.11
**Medical factors**					
Diabetes duration (years)	21.6 (12.8)		11.2 (12.8)		< 0.0001
Insulin use					< 0.0001
Yes		490 (100.0)		254 (22.1)	
Number of comorbidities (range 0-5)	1.1 (1.2)		1.6 (1.2)		< 0.0001
0		199 (40.6)		220 (19.2)	
1		138 (28.2)		329 (28.7)	
2		85 (17.3)		377 (32.9)	
3		44 (9.0)		147 (12.8)	
4		20 (4.1)		62 (5.4)	
5		4 (0.8)		12 (1.0)	
BMI (kg/m^2^)	26.2 (4.6)		29.1 (5.7)		< 0.0001
**Lifestyle factors**					
Smoking					0.81
Nonsmoking		457 (93.3)		1066 (92.9)	
Currently smoking		33 (6.7)		81 (7.1)	
Physical Activity (min/week)	184.4 (133.5)		179.2 (134.9)		0.48
Diet behavior (score 0-7)					
Diet-general diet	5.5 (1.5)		5.3 (1.7)		0.01
Diet-specific diet	5.1 (1.4)		5.3 (1.4)		0.05
Diet-spacing carbohydrates	5.6 (1.9)		5.3 (2.0)		0.002
**Quality of life**					
Health-related quality of life (score 0-100)	54.8 (26.9)		54.7 (25.7)		0.96
Life satisfaction (score 5-35)	16.2 (4.3)		16.6 (4.3)		0.14

BMI: body mass index

P value: t-tests/Chi-square analyses comparing differences between type 1 and type 2 diabetes samples.

### Determinants of HRQL in type 1 diabetes sample

In Model 1 (personal factors), older age (β = **-**0.11, p < 0.05), and higher activity trait (personality) scores (β = 0.38, p < 0.01) were significantly associated with a higher HRQL after controlling for other demographic factors. This model explained 17.4% of the variance for HRQL. In Model 2 (medical factors), a higher number of comorbidities (β = **-**0.31, p < 0.01) and a higher BMI (β = **-**0.16, p < 0.01) were associated with lower HRQL. This model explained 15.5% of the variance for HRQL. In Model 3, being a non-smoker (β = **-**0.14, p < 0.01), higher physical activity levels (β = 0.29, p < 0.01) and more days of spacing carbohydrates (β = 0.11, p < 0.05) were positively associated with HRQL. The model explained 10.6% of the variance for HRQL. In Model 4, higher activity trait (personality) scores (β = 0.28, p < 0.01), fewer comorbidities (β = **-**0.27, p < 0.01), lower BMI (β = **-**0.12, p < 0.01), currently non-smoking (β = **-**0.14, p < 0.01), and higher physical activity levels (β = 0.16, p < 0.01) were significantly associated with higher HRQL. This combined model explained 28.9% of the variance for HRQL. (Table 2)

**Table 2:** Results of multiple regression analysis for health-related quality of life and life satisfaction in adults with type 1 diabetes

	Model 1	Model 2	Model 3	Model 4
	β (HRQL/LS)	β (HRQL/LS)	β (HRQL/LS)	β (HRQL/LS)
**Personal factors**				
Age	**- **0.11*/0.09			0.02/0.10
Gender	0.03/0.08			**-**0.01/0.07
Marital status	0.06/0.12^†^			0.06/0.11*
Education	0.08/0.02			0.02/-0.01
Income	0.03/0.16^†^			**-**0.01/0.16^†^
Personality (activity trait)	0.38^†^/0.30^†^			0.28^†^/0.27^†^
**Medical factors**				
Diabetes duration		**-**0.07/0.06		**-**0.05/0.03
Insulin use		N/A		N/A
Number of comorbidities		**-**0.31^†^/**-**0.11^†^		**-**0.27^†^/**-**0.09
Body mass index		**-**0.16^†^/**-**0.09		**-**0.12^†^/**-**0.03
**Lifestyle factors**				
Smoking			-0.14^†^/0.07	**-**0.14^†^/**-**0.03
Physical activity			0.29^†^/0.08	0.16^†^/0.00
Diet-general			-0.04/0.06	**-**0.07/0.03
Diet-specific			0.03/0.08	0.02/0.06
Diet-spacing			0.11*/0.03	0.09/0.03
**Adjusted R^2 ^**	0.17^†^/0.13^†^	0.15^†^/0.02^†^	0.11^†^/0.03^†^	0.29^†^/0.14^†^

*p < 0.05, ^†^p < 0.01

HRQL: health-related quality of life, LS: life satisfaction

Smoking was coded: non-smoker = 0, current smoker = 1

### Determinants of life satisfaction in type 1 diabetes sample

In Model 1 (personal factors), having a partner (β = 0.12, p < 0.01), a higher income (β = 0.16, p < 0.01), and higher activity trait scores (β = 0.30, 1 < 0.01) were significantly associated with higher life satisfaction. The model explained 13.2% of variance for life satisfaction. In Model 2, number of comorbidities (β = **-**0.11, p < 0.01) was negatively associated with life satisfaction. This model explained 2.0% of the variance for life satisfaction. In model 3 (lifestyle behaviors) none of the variables were significantly associated with life satisfaction. The model explained 2.9% of variance for life satisfaction. In Model 4, marital status (β = 0.11, p < 0.05), income (β = 0.16, p < 0.01), and activity trait (β = 0.27, p < 0.01) remained significant. The combined model explained 14% of variance for life satisfaction. (Table 2)

### Interaction term with diabetes type

Factors significantly associated with HRQL (i.e., age, income, activity trait (personality), number of comorbidities, BMI, current smoking status, and physical activity) and life satisfaction (i.e., age, gender, marital status, income, activity trait (personality), number of comorbidities, BMI, current smoking status, and diet (general) score) in Model 4 were examined for interaction with diabetes type. The interaction of age and diabetes type was significant for HRQL (β = 0.05, p < 0.05, ɧ^2 ^= 0.016), Table 3). Advancing age was associated with increased HRQL in theT2D group, while age was inversely associated with HRQL in the T1D group. There were no significant interactions between the identified determinants and diabetes type in life satisfaction (Table 4).

**Table 3:** Interaction effects of diabetes type on determinants of health-related quality of life

Independent variable	Standardized Coefficients (β)	**Sig**.
T1D/T2D	0.08	0.0001
Age	0.07	0.01
Income	0.05	0.02
Activity trait (personality)	0.26	< 0.0001
Number of comorbidities	-0.24	< 0.0001
BMI	-0.20	< 0.0001
Smoking	-0.11	< 0.0001
Physical activity	0.13	< 0.0001
T1D/T2D × age	0.05	0.04
T1D/T2D × income	0.03	0.23
T1D/T2D × activity trait	-0.02	0.46
T1D/T2D × comorbidity	0.03	0.25
T1D/T2D × BMI	-0.02	0.40
T1D/T2D × smoking	0.02	0.31
T1D/T2D × physical activity	-0.02	0.29

T1D: type 1 diabetes, T2D: type 2 diabetes, BMI: body mass index

Smoking was coded: non-smoker = 0, current smoker = 1

**Table 4:** Interaction effects of diabetes type on determinants of life satisfaction

Independent variable	Standardized Coefficients (β)	**Sig**.
T1D/T2D	0.05	0.08
Age	0.15	< 0.001
Gender	0.07	0.005
Marital status	0.08	0.002
Income	0.12	< 0.001
Activity trait (personality)	0.26	< 0.001
Number of comorbidities	-0.10	< 0.001
BMI	-0.07	0.01
Smoking	-0.06	0.01
Diet (general)	0.10	< 0.001
T1D/T2D × age	0.05	0.08
T1D/T2D × gender	-0.003	0.88
T1D/T2D × marital status	-0.03	0.27
T1D/T2D × income	-0.02	0.43
T1D/T2D × activity trait	-0.01	0.71
T1D/T2D × comorbidity	-0.004	0.88
T1D/T2D × BMI	-0.01	0.58
T1D/T2D × smoking	-0.02	0.52
T1D/T2D × diet (general)	0.01	0.81

T1D: type 1 diabetes, T2D: type 2 diabetes, BMI: body mass index

Smoking was coded: non-smoker = 0, current smoker = 1

## Discussion

This study examined the differences in HRQL and life satisfaction scores between T1D and T2D groups, the determinants of HRQL and life satisfaction in adults with T1D, and interaction effects of diabetes type on identified determinants of HRQL and life satisfaction using data on a large sample of adults with diabetes. There were no differences in HRQL and life satisfaction scores between the two diabetes groups. We found that personality, numbers of comorbidities, BMI, smoking habits and physical activity were associated with HRQL, while demographic factors (marital status and income) and personality were associated with life satisfaction among adults with T1D. The only difference between the determinants of HRQL and life satisfaction between the two diabetes groups was age; the T2D group had a threshold association between advancing age and HRQL [11] compared to a negative linear relationship in the T1D group. The results of this study add to the limited literature on the determinants of HRQL and QoL in adults with T1D and on differences in determinants of HRQL and QoL between the two diabetes types.

Previous findings on the differences in HRQL scores between T1D and T2D groups have been mixed. In a study of 240 adults, the T2D group had higher HRQL compared to the T1D group, after adjusting for demographic factors (i.e., age, marital status and education), diabetes complications, and diabetes duration [24]. Another study (T1D N = 236, T2D N = 889) found a higher global health profile (SF-36) score in the T2D group compared to the T1D group [25]. In a survey of 1783 adults with diabetes, individuals with T1D had higher HRQL (physical functioning and social functioning) compared to those with T2D [44]. The same study reported no differences in HRQL between T1D and T2D patients treated by diet-only, but a lower HRQL score among T2D patients treated with insulin in comparison to T1D patients [44]. We did not observe significant differences in HRQL and life satisfaction scores between T1D and T2D groups; however, there were significant differences in a number of comorbidities and BMI, which were significantly associated with HRQL, in these two groups which may be explained by differences in the sample characteristics between the two diabetes groups.

The combined model, consisted of personal, medical and lifestyle factors, explained 29% and 14% of the variance respectively, for HRQL and life satisfaction, in our T1D sample which is comparable to our findings for the T2D samples (N = 1,147; 27% for HRQL and 18% for life satisfaction) [11]. Glasgow and colleagues [41] investigated HRQL and associated characteristics (demographic factors, medical factors, and self-care behaviors) in a large (N = 2,056) national sample of adults with diabetes, and found the explained variance to be 17% to 29% for three dimensions of HRQL (i.e., physical functioning, social functioning, and mental health) [44]. The study however, did not examine the factors separately for the T1D and T2D groups.

The variance explained by our model is lower compared to other studies that included psychosocial factors to explain HRQL in diabetes populations. Maddigan and associates [45] investigated factors associated with HRQL, and found that demographic, medical and psychosocial factors, (e.g., depression, stress, sense of belonging to the community, and perceived healthcare needs) were independently associated with HRQL; the model explained 36% of the variance for HRQL [45]. Another study examining coping style, diabetes-specific knowledge, doctor-patient relationship, personal characteristics, and illness on HRQL in adults with diabetes (T1D N = 224, T2D N = 401) reported an explained variance of 62% for HRQL [46]. The inclusion of psychosocial factors in a model has the potential to increase our understanding of HRQL and QoL, and may help identify relationships among psychological factors and other factors (demographics, personality, medical factors, and lifestyle behaviors).

In our study, demographic factors (i.e., marital status and income) were significantly associated with life satisfaction after controlling for other variables. This finding is consistent with previous research on non-diabetes populations [47,48]. Most T1D cases are diagnosed during childhood [23], and researchers have identified that pediatric diseases have negative effects on adulthood demographic factors (e.g., socioeconomic level, education, marital life) [49,50]. A review of studies on child-onset T1D identified that these individuals may have disadvantages in employment and are likely to have lower incomes in adulthood [51]. Our T1D sample demonstrated a lower annual income compared to the median income levels of the Alberta data from the 2005 Canadian Census [52]. Considering the observed significant, independent association of marital status and income with life satisfaction, support systems to improve these factors may improve QoL of T1D adults.

Personality (activity trait) was the strongest independent variable associated with HRQL and life satisfaction, which was consistent with our findings from the T2D group [11]. Although there is limited information on personality and HRQL in adults with T1D [53], the relationship between personality, HRQL and QoL is supported by the studies that identified relationships between personality and specific determinants of HRQL or QoL: glycemic control [54], diabetes complications [55,56], diabetes self-care behaviors [57], coping [58], mood [58] and social support [58]. The observed association of personality with HRQL and QoL in our study may be mediated by these determinants.

The inverse associations of BMI and comorbidities with HRQL are consistent with a previous study [19]. In 784 adults with T1D, BMI and comorbidities such as stroke, cardiovascular disease and high blood pressure were associated with reduced HRQL (Quality of Well Being index-SA health utility score) [19]. The positive relationship between physical activity and HRQL in our study was also consistent with research on 397 adults with T1D [18]. Although we could not identify any study that examined a direct relationship between smoking and HRQL in adults with T1D, smoking was associated with poor glycemic control [59] and renal complication [60], established determinants of HRQL in diabetes population.

Medical and lifestyle factors were not associated with life satisfaction, which was consistent with other studies [61,62]. In a general population study, BMI was significantly associated with HRQL but not with life satisfaction [61]. In a survey of 3,308 adults with/without chronic conditions, having a heart disease was associated with lower HRQL but not with rating of overall QoL, compared with healthy subjects [62].

We identified a significant interaction between age and diabetes type; however, the effect size was small according to the Cohen's guidelines [63]. The age distributions for the two diabetes groups (51.5 ± 16.4 years for T1D and 63.7 ± 11.4 years for T2D) may have influenced the effect of age on HRQL. The risk of poor self-rated health among diabetics was smaller in the older age group (60-74 years, odds ratio = 4.11, 95% CI = 2.91-5.80) compared to the younger age group (25-39 years, odds ratio = 16.10, 95% CI = 5.97-43.43)[64], suggesting that age could have different effects on HRQL between younger and older adults. The younger age of our T1D sample compared to T2D sample may have partially accounted for the age × diabetes type interaction.

There may also be psychosocial differences which could account for the age × diabetes type interaction. Studies have indicated that social support and its impact on HRQL are influenced by age. Among adults with chronic diseases, younger adults (18-44 years) reported lower social support compared to older adults (65 years and older) [65]. In a T2D sample, age was associated with better patient-provider relationships, and that better patient-provider relationship was associated with higher HRQL [66]. Having better social support among the older group may explain the positive relationship between age and HRQL in our T2D group. In addition, studies suggest poor social support among T1D individuals. A study of T1D adults with a history of pediatric diseases reported that these adults demonstrated delays or failure to achieve social development [67]. Also, among young adults, individuals with T1D showed poorer social support compared to a non-diabetic group [68]. More than 30% of our T1D sample was diagnosed with diabetes before the age of 18, which may have affected their social development and subsequent support.

Study strengths include a large population sample of adults with T1D and T2D adults, the use of validated measures of HRQL, life satisfaction and personality assessment. Several limitations however need to be acknowledged. First, because this was a secondary study, some measures were not specifically designed to examine HRQL or QoL. Further, as prior studies in diabetes population report determinants of HRQL vary for dimensions of HRQL [24,44], future studies are encouraged to test determinants of each specific component of HRQL. Second, the results cannot imply causality amongst the significant relationships because of cross-sectional data. To assess causality, intervention studies are needed to investigate whether intervening on the identified determinants could improve HRQL and QoL in adults with diabetes. Third, the study participants were recruited through Alberta Registry which may have resulted in more cases with T1D (30% of overall sample). Finally, our study didn't include other established determinants of HRQL and QoL (e.g., psychological factors, diabetes complications). Despite these limitations, our findings provide important information regarding the determinants of HRQL and QoL among T1D adults and the differences between the two diabetes populations.

The significant associations of medical and lifestyle factors with HRQL suggest that health practitioners should be encouraged to achieve good glycemic and cardiovascular risk factor control, and promote lifestyle interventions among T1D population. Demographic factors were significantly associated with life satisfaction in the T1D group. Previous studies have identified that diabetes, especially during earlier life, negatively affects socioeconomic status [50,51,69]. Our results imply that major health services targeting glycemic and cardiovascular risk factor control and lifestyle behaviors may not be sufficient to improve overall QoL of T1D adults. Additional support for socioeconomically disadvantaged individuals living with this disease may be warranted.

## Conclusions

In summary, medical factors and lifestyle behaviors were independently associated with HRQL in the T1D group. Health practitioners should be encouraged to achieve good glycemic and cardiovascular risk factor control, and promote lifestyle interventions to improve HRQL and overall QoL in this population. Additional support for socioeconomically disadvantaged adults with T1D may be needed. With the exception of age, the determinants of HRQL and QoL appear to be similar between T1D and T2D adults, suggesting that both diabetes groups may benefit from achieving generic, approaches in targeting optimal control of glycemic level and comorbidities as well as promoting healthy lifestyle.

## List of abbreviations

ALEXANDRA: Alberta Longitudinal Exercise and Diabetes Research Advancement; BMI: body mass index; CI: confidence interval; HRQL: health-related quality of life; QoL: quality of life; T1D: type 1 diabetes; T2D: type 2 diabetes.

## Competing interests

The authors declare that they have no competing interests.

## Authors' contributions

II performed data analysis, interpreted the data, and drafted the manuscript. RCP, KSC and JAJ were involved in study concept and design, acquisition of the data, data interpretation, manuscript drafting and revision of the manuscript. All authors approved the final manuscript.
